# Figure of Merit Enhancement of a Surface Plasmon Resonance Sensor Using a Low-Refractive-Index Porous Silica Film

**DOI:** 10.3390/s17081846

**Published:** 2017-08-10

**Authors:** Qing-Qing Meng, Xin Zhao, Cheng-You Lin, Shu-Jing Chen, Ying-Chun Ding, Zhao-Yang Chen

**Affiliations:** 1 College of Science, Beijing University of Chemical Technology, Beijing 100029, China; 2015200897@mail.buct.edu.cn (Q.-Q.M.); 2014200876@mail.buct.edu.cn (X.Z.); dingyc@mail.buct.edu.cn (Y.-C.D.); 2 School of Materials Science and Technology, China University of Geosciences (Beijing), Beijing 100083, China; chenshujing@cugb.edu.cn

**Keywords:** surface plasmon resonance sensor, figure of merit, porous silica, low-refractive-index

## Abstract

In this paper; the surface plasmon resonance (SPR) sensor with a porous silica film was studied. The effect of the thickness and porosity of the porous silica film on the performance of the sensor was analyzed. The results indicated that the figure of merit (FOM) of an SPR sensor can be enhanced by using a porous silica film with a low-refractive-index. Particularly; the FOM of an SPR sensor with 40 nm thick 90% porosity porous silica film; whose refractive index is 1.04 was improved by 311% when compared with that of a traditional SPR sensor. Furthermore; it was found that the decrease in the refractive index or the increase in the thickness of the low-refractive-index porous silica film can enlarge the FOM enhancement. It is believed that the proposed SPR sensor with a low-refractive-index porous silica film will be helpful for high-performance SPR sensors development.

## 1. Introduction

The surface plasmon resonance (SPR) sensor, as an important optical sensor, has been used in chemical and biochemical sensing, gases sensing, medical diagnostics, and food safety detection for years [1,2,3]. The rapid development of SPR sensor technology is owed to its distinctive features, such as label-free quantification, real-time analysis, and high sensitivity [4,5,6]. Among the various types of SPR sensors, one of the widely used geometries is the Kretschmann configuration which is generally prepared by evaporating a thin metal film on top of a glass prism. In this configuration, p-polarized light illuminates on the metal surface from the prism side to excite the surface plasmon wave (SPW) [7]. When the incident angle of the light source fulfills the SPR requirements, the propagation constant of incident light along the interface will match that of the SPW [8], causing the collective oscillation of electrons on the surface of the metal [9,10]. In this case, the energy of incident light transfers onto the surface plasmons, which leads to a significant decrease in the reflectance, and forms a narrow dip in the reflectance spectrum [11]. Reflectance dip shifts, caused by the change of the analyte refractive index, can be used for sensing. For instance, in the angular interrogation mode, a change in the analyte refractive index causes a shift in the resonance angle (at which the minimum reflectance is realized) for a given wavelength [12].

The figure of merit (FOM), defined as the ratio between the sensitivity (S) and the full width at half maximum (FWHM) of the reflectance dip, is a comprehensive parameter to evaluate the performance of an SPR sensor [5]. According to the definition of FOM, improving the sensitivity is an immediate way to achieve FOM enhancement. In recent years, many investigations have been carried out for sensitivity improvement. For example, Lahav et al. [13] facilitated sensitivity enhancement by using a guided-wave SPR structure, which was prepared by evaporating a 10 nm Si layer on top of the metal. Shukla et al. [14] and Bao et al. [15] both demonstrated that the sensitivity of an SPR sensor could be increased by adding a ZnO thin film. Besides, Benkabou et al. [16] achieved sensitivity enhancement by employing the dielectric multilayer structure in the SPR sensor.

Although it has been demonstrated that the addition of a thin dielectric layer with a high-refractive-index on top of the metal layer can improve the sensitivity of an SPR sensor [11], the FOM of the SPR sensor cannot be enhanced in this way. The reason is as follows: this sensitivity improvement method induces a significant broadening of the reflectance dip, which causes the decreasing of the FWHM, consequently leading to no change, or even a decrease in FOM [17]. Compared with high-refractive-index dielectric thin films, the low-refractive-index dielectric thin films seem to be seldom used for the performance improvement of an SPR sensor. In this paper, we studied the effect of a low-refractive-index dielectric thin film on the performance of an SPR sensor, which has not been reported in literature yet.

As an example of a study, porous silica (a kind of low-refractive-index material) was considered because its refractive index can change from 1.05 to 1.46 by controlling its porosity [18]. Besides having a low-refractive-index, porous silica film has several other advantageous properties, such as low thermal conductivity, high mechanical strength, and excellent thermal stability [19]. These characteristics make it applicable to many optical aspects, such as the preparation of antireflection coatings [20]. Particularly, porous silica film has also been employed for performance improvement of an SPR sensor by reducing its FWHM of the reflectance dip [21].

**Figure 1:**
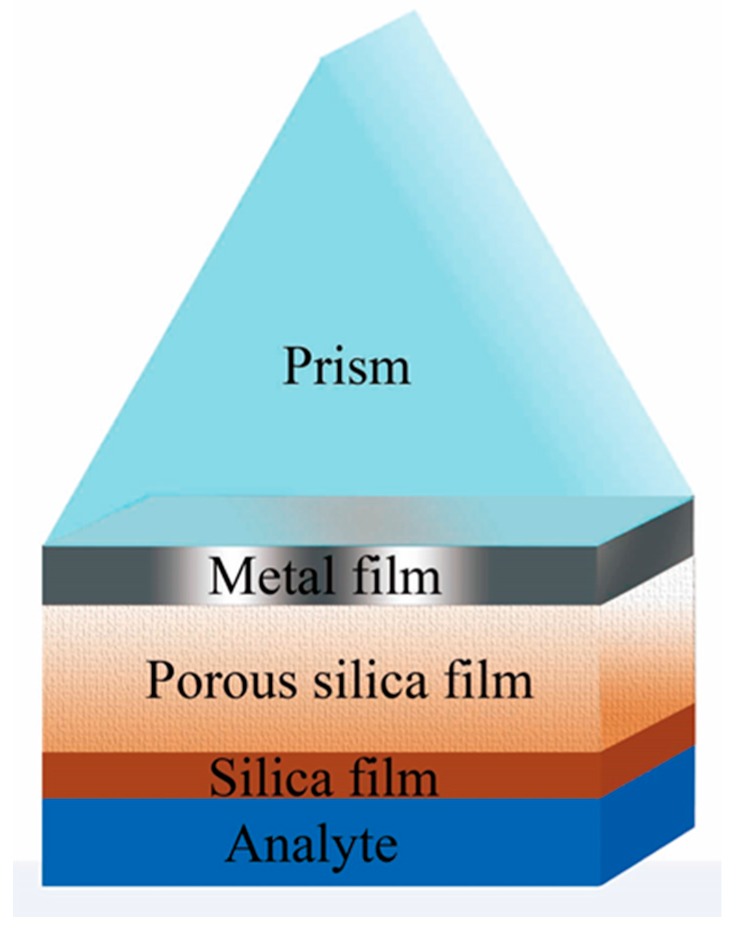
Schematic of the surface plasmon resonance (SPR) sensor with a porous silica film.

Therefore, it will be of much interest in this paper to study in detail the effect of a porous silica film on the performance of an SPR sensor, and to explore the possibility of FOM enhancement by using porous silica film. To prevent the diffusion of analyte fluid into the porous silica film, which may affect its refractive index, a thin SiO_2_ film was used as a protective film at top of the SPR sensor (as shown in Figure 1). The obtained result indicated that by adding a 40 nm thick porous silica film with the porosity of 90% (corresponding to the refractive index of 1.04) on top of the metal film, the FOM can be enhanced by an increase factor of 311%, compared with that of a traditional SPR sensor.

## 2. Calculation Methods

### 2.1. Three-Layer Model

**Figure 2:**
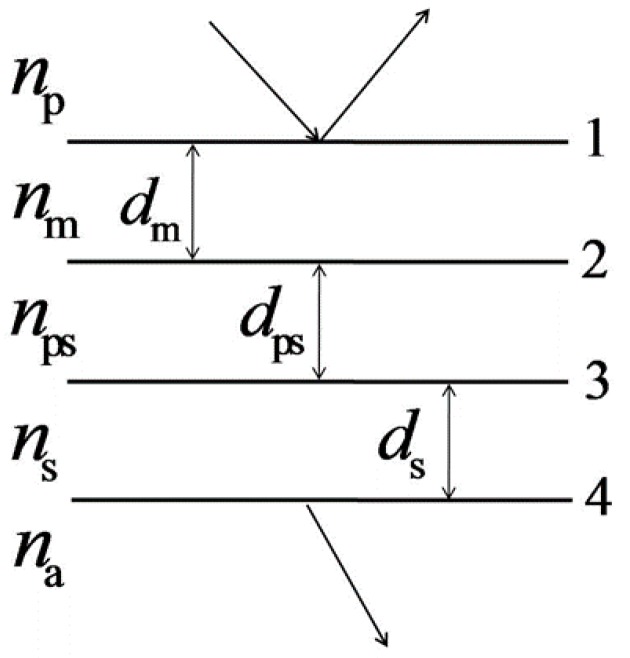
Three-layer model of the proposed SPR sensor.

To theoretically analyze the performance of an SPR sensor, we employed a typical thin-film theory [22], which has been widely validated with real SPR systems [23,24]. The SPR sensor with a porous silica film can be treated as a three-layer model (as shown in Figure 2), which contains a metal film, a porous silica film, and a silica film. The prism and analyte are considered as the incident and emergent media respectively.

### 2.2. Reflectance Coefficient

For this three-layer model, the tangential components of the electric and magnetic field amplitudes at interface 1 (*E*_1_ and *H*_1_) are connected to those at interface 4 (*E*_4_ and *H*_4_) by the characteristic matrix [22]. This is written as

(1) $$\left[\begin{array}{c} E_{1}\\ H_{1} \end{array}\right]=\left[\begin{array}{cc} \cos\delta_{m} & \frac{i}{\eta_{m}}\sin\delta_{m}\\ i\eta_{m}\sin\delta_{m} & \cos\delta_{m} \end{array}\right]\left[\begin{array}{cc} \cos\delta_{\mathrm{ps}} & \frac{i}{\eta_{\mathrm{ps}}}\sin\delta_{\mathrm{ps}}\\ i\eta_{\mathrm{ps}}\sin\delta_{\mathrm{ps}} & \cos\delta_{\mathrm{ps}} \end{array}\right]\left[\begin{array}{cc} \cos\delta_{s} & \frac{i}{\eta_{s}}\sin\delta_{s}\\ i\eta_{s}\sin\delta_{s} & \cos\delta_{s} \end{array}\right]\left[\begin{array}{c} E_{4}\\ H_{4} \end{array}\right],$$

where *δ*_i_ (i = m, ps, s) and *η*_i_ (i = m, ps, s) are the phase factor and the optical admittance of the metal (m), porous silica (ps), or silica (s) film. *δ*_i_ = 2π*n*_i_d_i_cos*θ*_i_/*λ*, in which *n*_i_ and *d*_i_ respectively represent the refractive index and thickness of each film. *λ* is the wavelength of the incident light, and *θ*_i_ represents the propagation angle in each medium. *η*_i_ = *n*_i_/cos*θ*_i_ is satisfied for p-polarized light.

Because *H*_4_/*E*_4_ = *η*_a_ (the optical admittance of the analyte) and *H*_1_/*E*_1_ = *Y* (the equivalent optical admittance of the assembly), Equation (1) can also be expressed as

(2) $$E_{1}\left[\begin{array}{c} 1\\ Y \end{array}\right]=\left[\begin{array}{cc} \cos\delta_{m} & \frac{i}{\eta_{m}}\sin\delta_{m}\\ i\eta_{m}\sin\delta_{m} & \cos\delta_{m} \end{array}\right]\left[\begin{array}{cc} \cos\delta_{\mathrm{ps}} & \frac{i}{\eta_{\mathrm{ps}}}\sin\delta_{\mathrm{ps}}\\ i\eta_{\mathrm{ps}}\sin\delta_{\mathrm{ps}} & \cos\delta_{\mathrm{ps}} \end{array}\right]\left[\begin{array}{cc} \cos\delta_{s} & \frac{i}{\eta_{s}}\sin\delta_{s}\\ i\eta_{s}\sin\delta_{s} & \cos\delta_{s} \end{array}\right]\left[\begin{array}{c} 1\\ \eta_{a} \end{array}\right]E_{4}.$$

So, the characteristic matrix of the assembly is

(3) $$\left[\begin{array}{c} B\\ C \end{array}\right]=\left[\begin{array}{cc} \cos\delta_{m} & \frac{i}{\eta_{m}}\sin\delta_{m}\\ i\eta_{m}\sin\delta_{m} & \cos\delta_{m} \end{array}\right]\left[\begin{array}{cc} \cos\delta_{\mathrm{ps}} & \frac{i}{\eta_{\mathrm{ps}}}\sin\delta_{\mathrm{ps}}\\ i\eta_{\mathrm{ps}}\sin\delta_{\mathrm{ps}} & \cos\delta_{\mathrm{ps}} \end{array}\right]\left[\begin{array}{cc} \cos\delta_{s} & \frac{i}{\eta_{s}}\sin\delta_{s}\\ i\eta_{s}\sin\delta_{s} & \cos\delta_{s} \end{array}\right]\left[\begin{array}{c} 1\\ \eta_{a} \end{array}\right].$$

The equivalent admittance *Y* can be calculated through *Y* = *C*/*B*, and the reflectance *R* of the SPR sensor is

(4) $$R=\left|\frac{\eta_{p}-Y}{\eta_{p}+Y}\right|,$$

where *η*_p_ is the optical admittance of the prism.

### 2.3. Performance Parameters

The performance of an SPR sensor can be described by several parameters, such as the resonance angle *θ*_res_, depth of dip *R*_res_ (the reflectance at the resonance angle), sensitivity, FWHM, and FOM. In the angular interrogation mode, the resonance angle *θ*_res_ changes with the refractive index of the analyte *n*_s_, so the sensitivity *S* can be expressed as the ratio between the resonance angle variation Δ*θ*_res_, and the variation of refractive index of the analyte Δ*n*_s_ as follows [5]:

(5) $$S=\frac{\Delta \theta_{\mathrm{res}}}{\Delta n_{s}}.$$

The FWHM can be determined by calculating the full width at half maximum of the reflectance dip (Δ*θ*_0.5_), expressed as

(6) $$\mathrm{FWHM}=\Delta \theta_{0.5}.$$

Thus, the value of the FOM can be calculated by

(7) $$\mathrm{FOM}=\frac{S}{\mathrm{FWHM}}.$$

## 3. Results and Discussions

The performance of a prism-based SPR sensor is generally determined by the wavelength and incident angle of the light source, the material and thickness of each film used in the SPR sensor, and the material of the prism and analyte [25]. In our simulations, a laser with 632.8 nm wavelength was taken as the incident light source. Silver (Ag) was applied as the metal material in the SPR sensor (*n*_m_ = 0.13 + 3.99i), which can provide sharper reflectance dip than gold (Au) [21,26]. The thickness of the Ag film was assumed to be 47 nm to ensure an approximate-zero reflectance at the resonance angle. As mentioned above, a 10 nm thick SiO_2_ film was employed as the protective film (*n*_s_ = 1.46). In addition, a 45-degree SF-11L glass was used as the prism (*n*_p_ = 1.73205) in the SPR sensor [27], and water was considered as the analyte (*n*_a_ = 1.33) in our simulations.

### 3.1. The Refractive Index of Porous Silica

**Figure 3:**
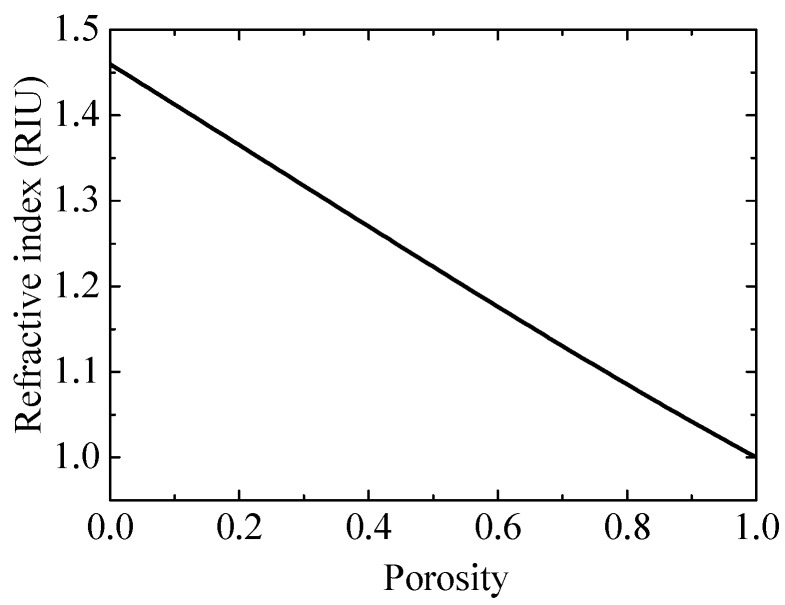
Refractive index as a function of the porosity of the porous silica film.

To determine the effect of a porous silica film on the performance of an SPR sensor, the characteristic of the refractive index of the porous silica film needs to be studied in advance. The two-component Bruggeman approximation model [20] can be adopted to express the relationship between the refractive index and the porosity for porous silica film, which is written as

(8) $$P\frac{1-n^{2}{}_{\mathrm{ps}}}{1+2n^{2}{}_{\mathrm{ps}}}+(1-P)\frac{n^{2}{}_{s}-n^{2}{}_{\mathrm{ps}}}{n^{2}{}_{s}+2n^{2}{}_{\mathrm{ps}}}=0,$$

where *P* is the porosity of the porous silica film, and *n*_s_ and *n*_ps_ are the refractive index of silica and porous silica, respectively. In this way, based on Equation (8), the relationship between the refractive index and the porosity can be determined, as shown in Figure 3.

### 3.2. The Effect of Porous Silica Film on the SPR Sensor

In our study, the porous silica films with a refractive index of 1.365, 1.270, 1.176, and 1.086, corresponding to 20%, 40%, 60%, and 80% porosity respectively, were chosen to explore the effect of a porous silica film on the performance of an SPR sensor.

#### 3.2.1. Porous Silica with 20% Porosity (*n*_ps_ = 1.365)

**Figure 4:**
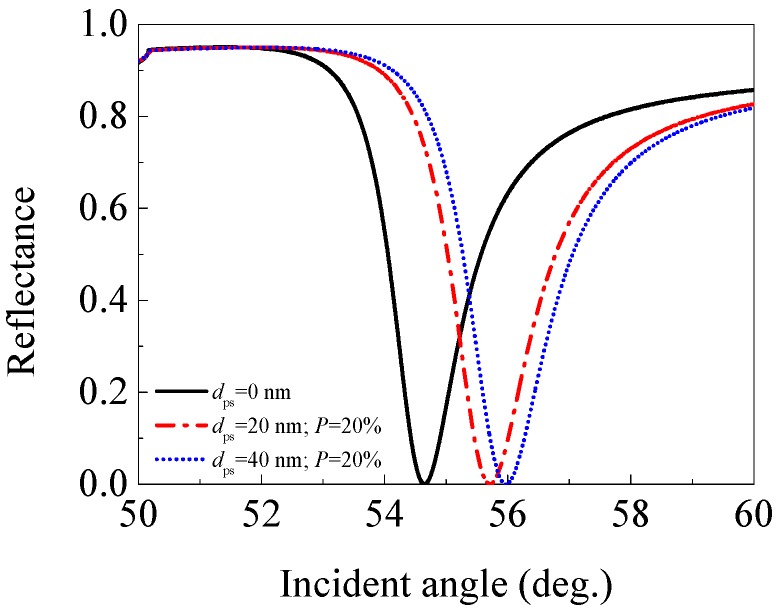
The angular spectra of the SPR sensors with 0 (traditional SPR sensor), 20, and 40 nm thick 20% porosity porous silica film (*n*_ps_ = 1.365).

Firstly, we presented the effect of a porous silica film with 20% porosity (*n*_ps_ = 1.365) on the performance of an SPR sensor. The angular spectra (reflectance versus incident angle) of the SPR sensors with 0 (traditional SPR sensor used for comparison), 20, and 40 nm thick 20% porosity porous silica film were plotted in Figure 4. The performance parameters for each sensor are listed in Table 1.

Compared with a traditional SPR sensor (without porous silica film, i.e., *d*_ps_ = 0 nm), the SPR sensor with a 20 nm (or 40 nm) thick 20% porosity porous silica film exhibits a slightly larger resonance angle and depth of dip (but *R*_res_ < 1 × 10^−3^). In addition, the sensitivity decreases from 67.9°/RIU for the traditional SPR sensor, to 53.7 (or 43.8)°/RIU for the SPR sensor with a 20 nm (or 40 nm) porous silica film, but the FWHM increases from 1.513° to 1.751° (or 1.784°). Thus, the FOM decreases from 44.888 RIU^−1^ to 30.654 (or 24.550) RIU^−1^. The results indicate that adding a 20 nm (or 40 nm) porous silica film with 20% porosity (*n*_ps_ = 1.365) in a traditional SPR sensor can decrease sensitivity and increase FWHM, which finally leads to the decreasing of the FOM.

#### 3.2.2. Porous Silica with 40% Porosity (*n*_ps_ = 1.270)

**Figure 5:**
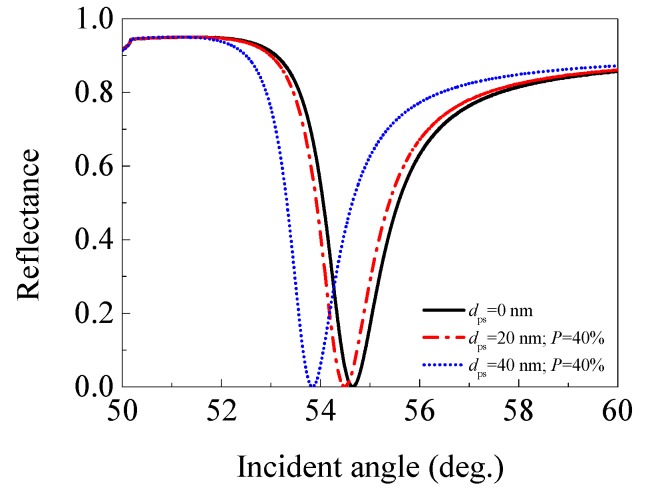
The angular spectra of the SPR sensors with 0 (traditional SPR sensor), 20, and 40 nm thick 40% porosity porous silica film (*n*_ps_ = 1.270).

Secondly, we showed the effect of a porous silica film with 40% porosity (*n*_ps_ = 1.270) on the performance of an SPR sensor. Figure 5 plotted the angular spectra of the SPR sensors with 0, 20, and 40 nm thick 40% porosity porous silica film, and Table 2 lists the performance parameters for each sensor.

Unlike the former case of porous silica film with 20% porosity, the SPR sensor with a 20 nm (or 40 nm) thick 40% porosity porous silica film exhibits a smaller resonance angle and depth of dip when compared with the traditional SPR sensor. Besides, the sensitivity decreases from 67.9°/RIU for the traditional SPR sensor, to 50.7 (or 41.5)°/RIU for the SPR sensor with a 20 nm (or 40 nm) porous silica film, with the FWHM also decreasing from 1.513° to 1.467° (or 1.320°). Consequently, the FOM decreases from 44.888 RIU^−1^ to 34.565 (or 31.443) RIU^−1^. The decreasing of the FOM should be due to a larger rate of decrease in the sensitivity (25.332% or 38.881%) than that of the FWHM (3.040% or 12.756%) when an additional 20 nm or 40 nm porous silica film with 40% porosity is employed. The results illustrate that adding a 20 nm (or 40 nm) porous silica film, with 40% porosity (*n*_ps_ = 1.270) in a traditional SPR sensor can cause the decrease in sensitivity, FWHM, and FOM.

#### 3.2.3. Porous Silica with 60% Porosity (*n*_ps_ = 1.176)

**Figure 6:**
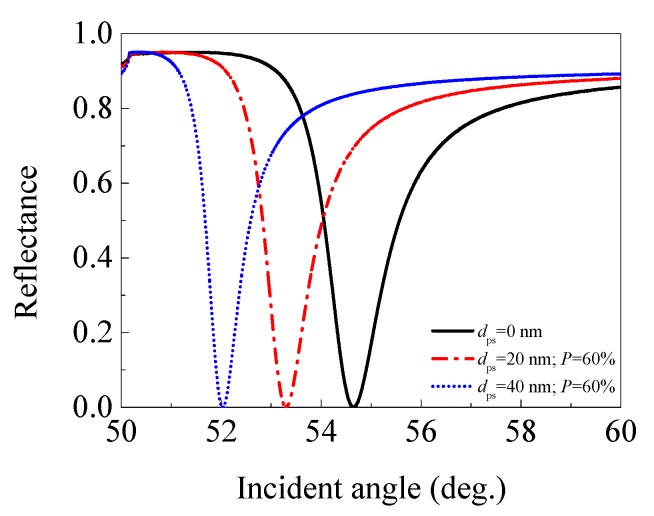
The angular spectra of the SPR sensors with 0 (traditional SPR sensor), 20, and 40 nm thick 60% porosity porous silica film (*n*_ps_ = 1.176).

Thirdly, the effect of a porous silica film with 60% porosity (*n*_ps_ = 1.176) on the performance of an SPR sensor was presented. The angular spectra of the SPR sensors with 0 (traditional SPR sensor), 20, and 40 nm thick 60% porosity porous silica film were plotted in Figure 6, and the performance parameters for each sensor are listed in Table 3.

The SPR sensor with a 20 nm (or 40 nm) thick 60% porosity porous silica film still exhibits a smaller resonance angle and depth of dip than the traditional SPR sensor without porous silica film (*d*_ps_ = 0 nm). The sensitivity decreases from 67.9°/RIU for the traditional SPR sensor, to 48.1 (or 40.8)°/RIU for the SPR sensor with a 20 nm (or 40 nm) porous silica film, and the FWHM decreases from 1.513° to 1.195° (or 0.910°). Consequently, the FOM decreases from 44.888 RIU^−1^ to 40.245 (or 44.129) RIU^−1^. The decreasing of the FOM still comes from the larger rate of decrease in the sensitivity (29.161% or 39.912%) than that of the FWHM (21.018% or 39.855%) when an additional 20 nm or 40 nm porous silica film with 60% porosity is used.

#### 3.2.4. Porous Silica with 80% Porosity (*n*_ps_ = 1.086)

**Figure 7:**
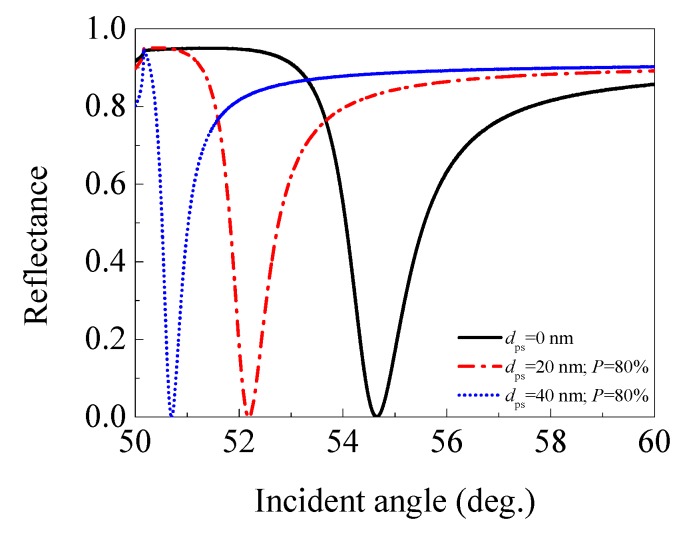
(Color online) The angular spectra of the SPR sensors with 0 (traditional SPR sensor), 20, and 40 nm thick 80% porosity porous silica film (*n*_ps_ = 1.086).

Finally, we presented the effect of a porous silica film with 80% porosity (*n*_ps_ = 1.086) on the performance of an SPR sensor. The angular spectra of the SPR sensors with 0 (traditional SPR sensor used for comparison), 20, and 40 nm thick 80% porosity porous silica film were given in Figure 7, and the performance parameters for each sensor are listed in Table 4.

Similar to the case of 60% porosity porous silica film, the SPR sensor with a 20 nm (or 40 nm) thick 80% porosity porous silica film also exhibits a smaller resonance angle and depth of dip than a traditional SPR sensor (*d*_ps_ = 0 nm). The sensitivity decreases from 67.9°/RIU for the traditional SPR sensor, to 46.2 (or 42.9)°/RIU for the SPR sensor with a 20 nm (or 40 nm) thick 80% porosity porous silica film, while the FWHM decreases from 1.513° to 0.934° (or 0.505°). Surprisingly, the FOM does not decrease, but increases from 44.888 RIU^−1^ to 49.646 (or 84.866) RIU^−1^. The reason for the increasing of the FOM should be attributed to the smaller rate of decrease in the sensitivity (31.959% or 36.819%) than that of the FWHM (38.268% or 66.623%) when an additional 20 nm or 40 nm porous silica film with 80% porosity is applied. This reflects the fact that adding a 20 nm (or 40 nm) porous silica film with 80% porosity (*n*_ps_ = 1.086) in a traditional SPR sensor can realize FOM enhancement.

#### 3.2.5. Summary

Based on the investigation results, we conclude that by adding a 20 nm or 40 nm porous silica film with 20%, 40%, or 60% porosity in a traditional SPR sensor, the FOM of the sensor could not be enhanced. However, by adding a 20 nm or 40 nm porous silica film with 80% porosity, the FOM of the sensor can be significantly improved. The enhanced FOM can be as high as 1.89 times that of a traditional SPR FOM by using a 40 nm thick 80% porosity porous silica film. One can notice that whether the FOM may be enhanced or not depends on the porous silica film’s porosity and thickness. It is worth exploring how the FOM varies with the refractive index (or porosity) and thickness of porous silica film.

#### 3.2.6. Origin of FOM Enhancement

**Figure 8:**
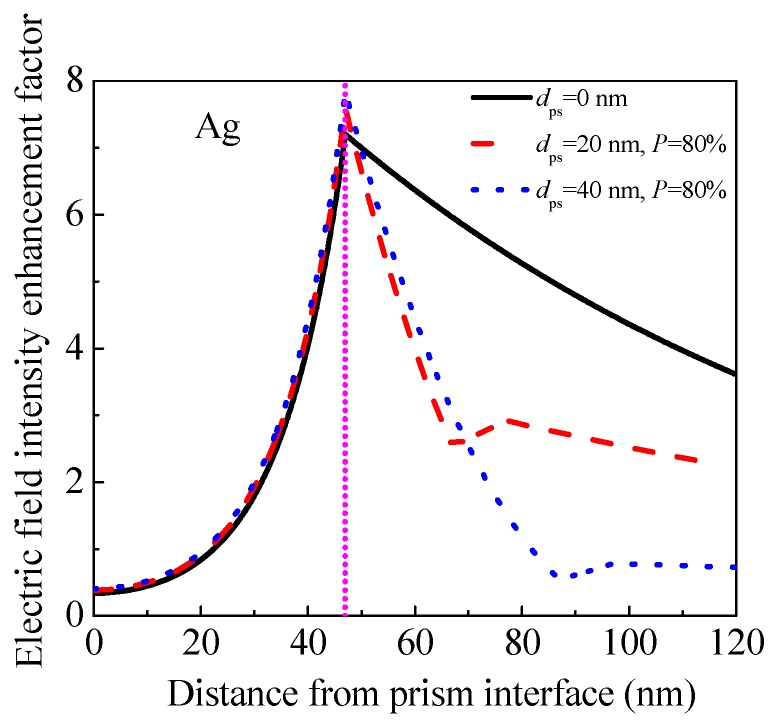
(Color online) Electric field intensity enhancement factor inside the SPR sensors in Figure 7.

To further investigate the origin of FOM enhancement achieved by adding a 20 nm or 40 nm porous silica film with 80% porosity porous silica film in a traditional SPR sensor, we calculated the electric field intensity enhancement factor inside the SPR sensors as discussed in Figure 7. We utilized the method described by Shalabney and Abdulhalim [28], as shown in Figure 8. When adding a 20 nm (or 40 nm) porous silica film with 80% porosity, the electric field intensity enhancement factor at the analyte interface inside the SPR sensor decreases from 7.20 to 2.92 (or 0.78), which is considered to be the reason for the decrease in sensitivity according to Reference [28]. Meanwhile, the maximum value of the electric field intensity enhancement factor increases from 7.20 to 7.60 (or 7.84), which is the origin of the decrease in FWHM as demonstrated by Chen et al. [29], and should be responsible for FOM enhancement.

### 3.3. The Effect of a Porous Silica Film on an SPR Sensor

**Figure 9:**
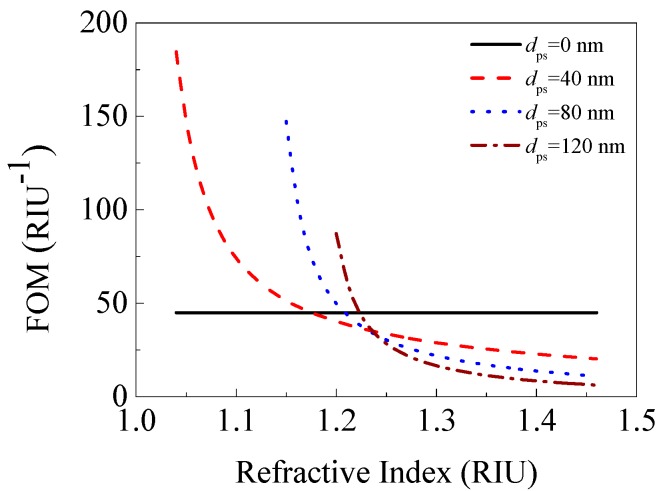
FOM versus the refractive index of porous silica film with various thicknesses.

We plotted the curves of the FOM versus the refractive index of the porous silica film with various thicknesses in Figure 9. Here, the porous silica films with 0 (traditional SPR sensor), 40, 80, and 120 nm thicknesses were considered. The results for only low depth of dip (*R*_res_ < 1 × 10^−2^) were shown in Figure 9.

In Figure 9, except for the FOM curve for *d*_ps_ = 0 nm (traditional SPR sensor used for comparison), other FOM curves all go up with the decreasing of the refractive index of porous silica film. This indicates that the porous silica film with a lower refractive index (i.e., higher porosity) can realize a larger FOM. In addition, the FOM enhancement can be realized when the refractive index of the porous silica film is less than a critical value for a given film thickness. The critical refractive index is 1.162, 1.202, and 1.219 for 40, 80, and 120 nm thick porous silica film, respectively. Furthermore, the FOM enhancement increases with the thickness of the low-refractive-index porous silica film. However, with the increasing of the thickness, the depth of dip also rapidly increases, which degrades the signal-to-noise of the SPR sensor [11]. Under the condition of low depth of dip (*R*_res_ < 1 × 10^−2^), the largest FOM enhancement is achieved by adding a 40 nm thick porous silica film with 90% porosity (*n*_ps_ = 1.04) in a traditional SPR sensor, which can enhance the FOM by an increase factor of 311%.

## 4. Conclusions

The SPR sensor with a porous silica film has been theoretically investigated in this paper. Compared with a traditional SPR sensor, the SPR sensor with a porous silica film exhibited higher FOM when the refractive index of porous silica film is less than a critical value for a given film thickness. Particularly, the addition of a 40 nm porous silica film with 90% porosity (*n*_ps_ = 1.04) in a traditional SPR sensor can improve the FOM by an increase factor of 311%. The FOM enhancement is due to the smaller rate of decrease in the sensitivity than that of the FWHM when an additional low-refractive-index porous silica film is employed. In addition, the FOM enhancement becomes larger when the refractive index of a porous silica film becomes lower, or the thickness of the low-refractive-index porous silica film becomes larger. Although only porous silica film has been studied as an example in this paper, the principles and conclusions of FOM enhancement are also applicable to other low-refractive-index materials. This research provides a way for improving the FOM of a prism-based SPR sensor, and may be useful for high-performance SPR sensors development.

## Figures and Tables

**Table 1:** The performance parameters of the SPR sensors with 0 (traditional SPR sensor), 20, and 40 nm thick 20% porosity porous silica film (*n*_ps_ = 1.365).

Thickness of Porous Silica Film (nm)	Resonance Angle (°)	Depth of Dip	Sensitivity (°/RIU)	FWHM (°)	FOM (RIU^−1^)
0	54.650	3.27 × 10^−4^	67.9	1.513	44.888
20	55.715	3.58 × 10^−4^	53.7	1.751	30.654
40	55.974	3.65 × 10^−4^	43.8	1.784	24.550

**Table 2:** The performance parameters of the SPR sensors with 0 (traditional SPR sensor), 20, and 40 nm thick 40% porosity porous silica film (*n*_ps_ = 1.270).

Thickness of Porous Silica Film (nm)	Resonance Angle (°)	Depth of dip	Sensitivity (°/RIU)	FWHM (°)	FOM (RIU^−1^)
0	54.650	3.27 × 10^−4^	67.9	1.513	44.888
20	54.493	3.22 × 10^−4^	50.7	1.467	34.565
40	53.837	3.00 × 10^−4^	41.5	1.320	31.443

**Table 3:** The performance parameters of the SPR sensors with 0 (traditional SPR sensor), 20, and 40 nm thick 60% porosity porous silica film (*n*_ps_ = 1.176).

Thickness of Porous Silica Film (nm)	Resonance Angle (°)	Depth of Dip	Sensitivity (°/RIU)	FWHM (°)	FOM (RIU^−1^)
0	54.650	3.27 × 10^−4^	67.9	1.513	44.888
20	53.299	2.82 × 10^−4^	48.1	1.195	40.242
40	52.034	2.39 × 10^−4^	40.8	0.910	44.129

**Table 4:** The performance parameters of the SPR sensors with 0 (traditional SPR sensor), 20, and 40 nm thick 80% porosity porous silica film (*n*_ps_ = 1.086).

Thickness of Porous Silica Film (nm)	Resonance Angle (°)	Depth of Dip	Sensitivity (°/RIU)	FWHM (°)	FOM (RIU^−1^)
0	54.650	3.27 × 10^−4^	67.9	1.513	44.888
20	52.189	2.44 × 10^−4^	46.2	0.934	49.464
40	50.701	1.94 × 10^−4^	42.9	0.505	84.966
